# Biliary stenting as a bridge to cholecystectomy in patients with choledocholithiasis

**DOI:** 10.1007/s00464-026-13260-z

**Published:** 2026-08-21

**Authors:** David Pinto, David Farkas, Lena Anthuber, Dmytro Vlasenko, Florian Sommer, Matthias Schrempf, Franz Poch, Christoph Römmele, Johannes Lauscher, Helmuth Messmann, Katharina Beyer, Michael Hoffmann

**Affiliations:** 1 Department of General, Visceral, Transplant and Thoracic Surgery, University Hospital Augsburg, Stenglinstraße 2, 86156 Augsburg, Germany; 2 Department of Gastroenterology and Infectiology, University Hospital Augsburg, Augsburg, Germany

**Keywords:** Choledocholithiasis, Biliary stenting, Cholecystectomy, ERCP, Recurrent biliary events

## Abstract

**Background:**

Early laparoscopic cholecystectomy after endoscopic retrograde cholangiopancreatography (ERCP) is recommended for patients with choledocholithiasis to reduce the risk of recurrent biliary events (RBE). However, in clinical practice early surgery is not always feasible. The role of biliary stenting as a bridging strategy before delayed cholecystectomy remains insufficiently studied. This study evaluated outcomes of delayed cholecystectomy after ERCP with biliary stent placement compared with early surgery in a stented cohort.

**Methods:**

We conducted a retrospective single-center study including patients who underwent ERCP with biliary stent placement followed by early cholecystectomy (≤ 14 days; eCCY) or delayed elective cholecystectomy (> 14 days; dCCY). The primary endpoint was the occurrence of RBE. Inverse probability of treatment weighting (IPTW) and sensitivity analysis were performed to address baseline imbalances. Multivariable logistic regression was performed to identify independent predictors of RBE. Incidence rates per person-time were calculated to account for differences in exposure time. Kaplan–Meier analysis assessed the temporal distribution of events. A sensitivity analysis was conducted excluding patients with acute cholecystitis at presentation.

**Results:**

A total of 338 patients were included (85 eCCY, 253 dCCY). Overall RBE occurred in 16.9% of patients, with no significant difference between groups (20.0 vs. 15.8%, *p* = 0.372). Timing of cholecystectomy was not independently associated with RBE (odds ratio 1.40, 95% confidence interval 0.71–2.76, *p* = 0.340). The incidence rate was 3.04 per 100 person-days in the eCCY group and 0.22 in the dCCY group, reflecting non-random treatment allocation. Intraoperative and postoperative outcomes were comparable. However, patients requiring urgent surgery due to RBE demonstrated increased operative complexity. The IPTW adjustment and sensitivity analysis confirmed all primary findings.

**Conclusions:**

In selected patients undergoing ERCP with biliary stent placement, delayed cholecystectomy was safe and feasible and was not associated with worse perioperative outcomes compared with early surgery. RBE occurred predominantly in the early post-ERCP period, but overall risk was not increased by delayed surgery. Patients requiring urgent surgery represent a higher-risk subgroup.

**Supplementary Information:**

The online version contains supplementary material available at https://doi.org/10.1007/s00464-026-13260-z.

Choledocholithiasis is a common complication of gallstone disease and represents a frequent indication for endoscopic and surgical intervention worldwide. Endoscopic retrograde cholangiopancreatography (ERCP) with endoscopic sphincterotomy and stone extraction is an established and highly effective method for clearance of common bile duct stones, achieving success rates exceeding 90% [1]. Definitive management, however, requires subsequent cholecystectomy as omission or delay of gallbladder removal is associated with a significant risk of recurrent biliary events (RBE), including biliary colic, acute cholecystitis, cholangitis, and pancreatitis [2–5].

The optimal timing of laparoscopic cholecystectomy following ERCP remains a matter of ongoing debate. The timing of cholecystectomy is commonly categorized as early, delayed, or on demand. However, the definitions of early and delayed vary considerably across studies. Overall, the available evidence favors early laparoscopic cholecystectomy. Retrospective studies, randomized controlled trials and meta-analyses have demonstrated that early cholecystectomy is associated with significantly lower risk of RBE, lower conversion rates, and reduced overall morbidity compared with delayed surgery [6–8]. On the basis of this evidence, the European Society of Gastrointestinal Endoscopy (ESGE) recommends performing laparoscopic cholecystectomy within two weeks after ERCP for choledocholithiasis, with a preference for early intervention when feasible [1].

Despite these recommendations, real-world clinical practice often deviates from guideline-based timing. Organizational constraints, patient comorbidities, interdepartmental logistics, and limited operative capacity frequently lead to delayed or elective cholecystectomy after successful biliary decompression. In this context, temporary biliary stent placement during ERCP is commonly employed to ensure sustained bile drainage, particularly in cases with incomplete stone clearance, difficult anatomy, or significant inflammation [1]. While biliary stenting is well established as a bridge to definitive clearance of the bile duct, its impact on the safety of delayed cholecystectomy has not been adequately addressed in the existing literature.

Importantly, most previous studies evaluating the timing of cholecystectomy after ERCP have analyzed heterogeneous patient populations, often combining patients with and without biliary stent placement. Consequently, the specific subgroup of patients who undergo ERCP with systematic biliary stenting remains underrepresented, despite the fact that these patients constitute a substantial proportion of daily clinical practice. Whether the presence of a biliary stent mitigates the risks associated with delayed cholecystectomy such as RBE, increased operative difficulty or longer hospital stay remains unclear.

The present study aims to address this knowledge gap by focusing exclusively on patients with choledocholithiasis who underwent ERCP with biliary stent placement. By comparing outcomes between patients who underwent early cholecystectomy during the first two weeks and those managed with an elective, delayed surgical approach, we sought to evaluate the safety and feasibility of deferred cholecystectomy in this specific setting.

By providing real-world data from a homogeneous cohort of stented patients, this study aims to contribute to evidence-based decision-making and to inform individualized treatment strategies in the management of choledocholithiasis.

## Materials and methods

### Reporting guidelines

This study was reported in accordance with the STROBE guidelines for observational studies.

### Study population

This retrospective single-center study was conducted at the Department of General Surgery, Visceral, Transplant and Thoracic Surgery at the University Hospital Augsburg in Germany. We reviewed the medical records of all consecutive patients admitted between January 2018 and December 2023 with confirmed choledocholithiasis who underwent endoscopic retrograde cholangiopancreatography (ERCP) with successful biliary clearance followed by biliary stent placement.

Only patients who subsequently underwent laparoscopic cholecystectomy at our institution were included in the analysis. Patients with incomplete medical records or insufficient follow-up data were excluded.

Demographic characteristics, clinical presentation, laboratory values, procedural details, operative findings, and postoperative outcomes were extracted from electronic medical records.

### Therapeutic strategies and definitions

In all included patients, a straight temporary plastic biliary stent was placed after endoscopic sphincterotomy and complete clearance of the common bile duct during ERCP to ensure adequate biliary drainage. The size of the plastic biliary stent was at the discretion of the endoscopist and ranged from 7 to 10 Fr. Stent length, stone size, stone number, and common bile duct diameter were not systematically documented in the endoscopy reports and could therefore not be included in the analysis.

Based on the interval between ERCP and cholecystectomy, patients were divided into two groups:

Patients who underwent laparoscopic cholecystectomy within two weeks after ERCP and were treated in accordance with current European guideline recommendations, formed the early elective cholecystectomy group (eCCY).

Patients in whom elective laparoscopic cholecystectomy was scheduled more than two weeks after ERCP, using biliary stenting as a bridging strategy to delayed surgery, formed the delayed cholecystectomy group (dCCY).

We performed a subgroup analysis comparing patients undergoing urgent unplanned cholecystectomy triggered by a recurrent biliary event with patients undergoing early cholecystectomy within two weeks after ERCP. Urgent surgery was defined as cholecystectomy performed during an unplanned admission due to RBE.

Stent dwell time was extracted from the available endoscopy reports. It was defined as the interval between ERCP with documented common bile duct clearance and biliary stent placement and documented stent removal.

### Study endpoints and definition of outcomes

The primary endpoint of this study was the occurrence of recurrent biliary events (RBE) during the interval between ERCP and cholecystectomy. Recurrent biliary events (RBE) were defined as any emergency department visit or unplanned hospitalization related to biliary pathology occurring after ERCP and before cholecystectomy. These included symptomatic stent dysfunction, biliary colic due to cholelithiasis, acute cholecystitis, recurrent choledocholithiasis, acute cholangitis, or biliary pancreatitis.

Secondary endpoints included perioperative and postoperative outcomes, specifically: operative time, rate of conversion to open surgery, intraoperative complications and blood loss, postoperative complications, length of postoperative hospital stay, total length of hospital stay and overall morbidity and mortality within 30 days after surgery.

Conversion to open surgery was defined as any intraoperative change from laparoscopic to open cholecystectomy.

Postoperative complications were graded according to the Clavien–Dindo classification [9]. Clinically relevant complications were defined as Clavien–Dindo grade ≥ III.

Postoperative length of stay was defined as the number of days from the date of surgery to hospital discharge.

Total length of hospital stay was defined as the cumulative number of inpatient days related to biliary disease from the index ERCP admission until discharge after cholecystectomy. This included all hospitalizations occurring during the bridging interval, such as readmissions due to recurrent biliary events or stent-related complications.

### Statistical analysis

Continuous variables were tested for normal distribution using the Shapiro–Wilk test. Normally distributed variables were presented as mean ± standard deviation and compared using the Student’s t-test. Non-normally distributed variables were expressed as median with interquartile range and compared using the Mann–Whitney *U* test.

Categorical variables were presented as frequencies and percentages and compared using the chi-square test or Fisher’s exact test, as appropriate.

To identify independent predictors of RBE, a multivariable logistic regression analysis was performed including clinically relevant variables selected based on known association with biliary disease severity and treatment allocation. These variables included age, acute cholecystitis, cholangitis, biliary pancreatitis at the time of presentation and timing of cholecystectomy (early vs delayed). Odds ratios (ORs) with 95% confidence intervals (CI) were reported.

To address potential confounding by indication, inverse probability of treatment weighting (IPTW) was performed. A propensity score was estimated using logistic regression with group assignment (eCCY vs. dCCY) as the dependent variable and all baseline covariates as predictors. Stabilized inverse probability weights were calculated to create a pseudo-population in which baseline covariates were balanced between groups. Covariate balance before and after weighting was assessed using standardized mean differences (SMD), with an SMD < 0.1 considered indicative of adequate balance. The primary and secondary outcomes were then re-analyzed in the IPTW-weighted cohort.

Given the substantial difference in observation time between groups, RBE incidence rates were calculated as the number of events per 100 person-days of observation for each group. The observation period was defined as the interval between bile duct clearance with stent implantation during ERCP and either the occurrence of the first RBE or cholecystectomy, whichever occurred first. Cumulative person-days were calculated for each group and the incidence rates were compared descriptively.

The temporal distribution of RBE was described using the Kaplan–Meier method, with comparisons between groups assessed by the log-rank test. The time interval was defined as the period between duct clearance with stent implantation during ERCP and the occurrence of a RBE. Patients who underwent cholecystectomy without experiencing a RBE were censored at the time of surgery. A supplementary multivariable Cox proportional hazards regression analysis was performed and is reported in the Supplementary Material.

Sensitivity analyses were performed to assess the robustness of the findings. First, the primary analysis was repeated after exclusion of all patients presenting with acute cholecystitis at initial diagnosis. Second, intraoperative and postoperative outcomes were re-analyzed in this restricted cohort. Third, an additional sensitivity analysis used an objective RBE definition excluding pain-only events. This analysis was conducted in the unweighted cohort, the IPTW-weighted cohort, and the sensitivity cohort excluding patients with acute cholecystitis.

A two-sided *p*-value < 0.05 was considered statistically significant. Statistical analyses were performed by the main author using SPSS software (IBM SPSS Statistics, version 31, IBM Corp., Armonk, NY, USA).

No formal sample size calculation was performed as this was a retrospective analysis of all consecutive patients meeting the inclusion criteria during the study period.

### Ethical approval

This study was conducted in accordance with the principles of the Declaration of Helsinki. The study protocol was reviewed and approved by the institutional ethics committee of the Ludwig Maximilians University, Munich, Germany (approval number: 24–0980). Due to the retrospective study design and anonymized data analysis, the requirement for informed consent was waived.

### AI disclosure

During the preparation of this work, the authors used large language models (Claude) to support language editing. After using these tools, the authors reviewed and edited the content as needed and take full responsibility for the content of the published article.

## Results

### Study population

We reviewed the medical records of 338 patients diagnosed with choledocholithiasis who underwent ERCP with biliary stent placement at our institution between 2018 and 2023.

**Fig. 1:**
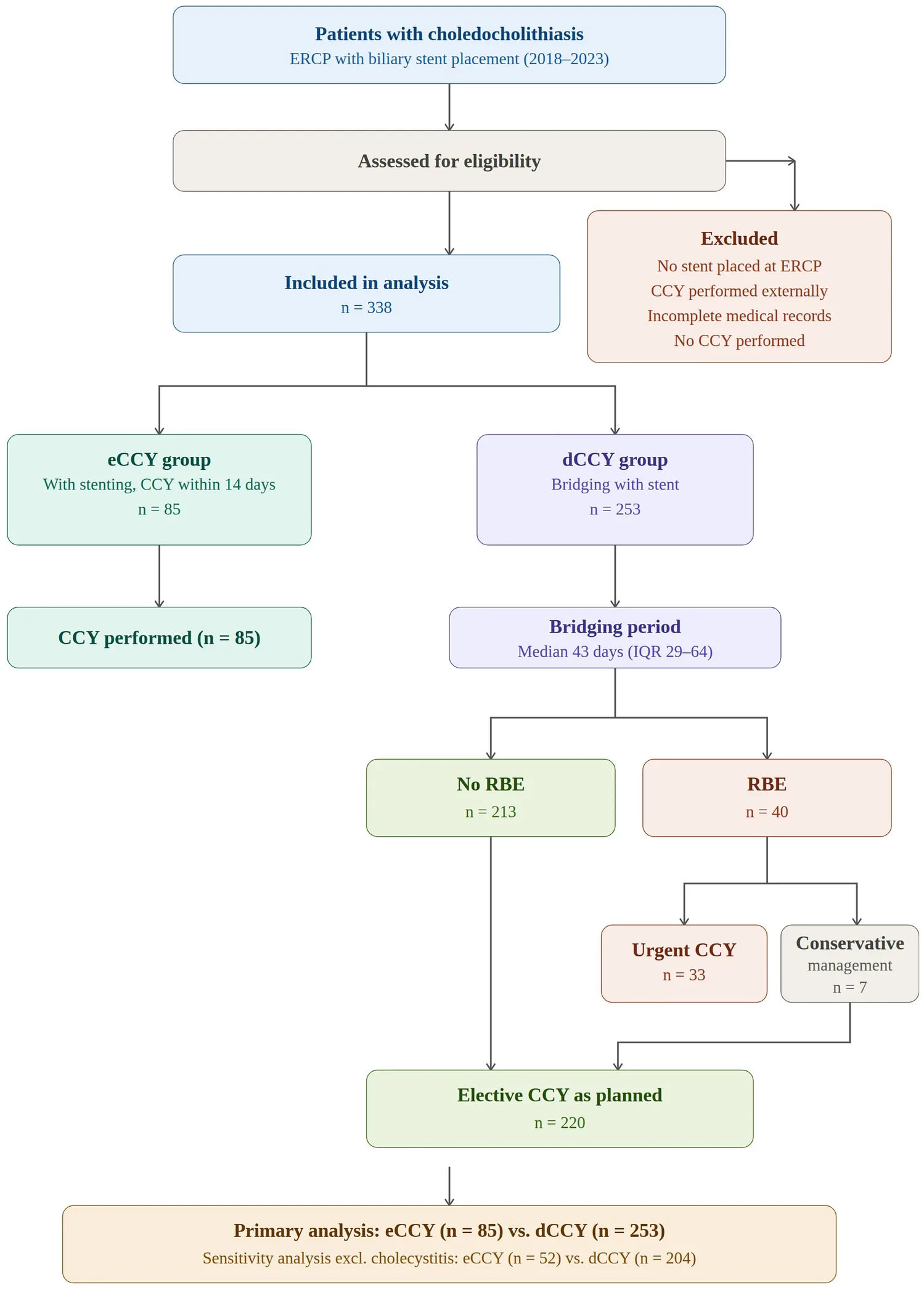
Study flowchart. *eCCY* early cholecystectomy (≤ 14 days after ERCP); *dCCY* delayed cholecystectomy (> 14 days after ERCP), *CCY* cholecystectomy, *ERCP* endoscopic retrograde cholangiopancreatography, *RBE* recurrent biliary event

The study flowchart illustrating patient selection, group allocation, and outcomes is presented in Fig. 1. A total of 338 patients were included in the analysis, of whom 85 underwent early cholecystectomy within 14 days (eCCY) and 253 were scheduled for delayed cholecystectomy with biliary stenting as a bridging strategy (dCCY). During the bridging period, 40 patients (15.8%) in the dCCY group developed RBE, of whom 33 required urgent cholecystectomy and 7 were managed conservatively before undergoing elective cholecystectomy as planned. Overall, 220 patients (86.9%) in the dCCY group underwent elective cholecystectomy without experiencing a RBE or after successful conservative management.

Baseline characteristics of the study population are summarized in Table 1. There were no statistically significant differences in demographic characteristics between the two groups (Table 1). At the time of diagnosis, 38.8% (*n* = 33) of patients in the eCCY group presented with concomitant acute cholecystitis compared with 19.4% (*n* = 49) in the dCCY group (*p* = 0.001). Conversely, biliary pancreatitis at presentation was significantly more frequent in the dCCY group compared with the eCCY group (24.5 vs. 11.8%, *p* = 0.013). There was no significant difference between the groups regarding the rate of concomitant cholangitis (Table 1).

**Table 1:** Baseline characteristics of the study population and comparison between groups

Characteristics	Overall (*n* = 338)	eCCY (*n* = 85)	dCCY (*n* = 253)	*p*-value
Demographics				
Age, years, mean ± SD	64.8 ± 16.7	63.6 ± 17.7	65.3 ± 16.4	0.443
Male, *n* (%)	163 (48.2)	48 (56.5)	115 (45.5)	0.079
Female, *n* (%)	175 (51.8)	37 (43.5)	138 (54.5)	
BMI ≥ 30 kg/m^2^, *n* (%)	92 (27.2)	24 (28.2)	68 (26.9)	0.808
ASA score, *n* (%)				
1	47 (13.9)	15 (17.6)	32 (12.6)	0.249
2	158 (46.7)	38 (44.7)	120 (47.4)	0.663
3	125 (37.0)	29 (34.1)	96 (37.9)	0.527
4	8 (2.4)	3 (3.5)	5 (2.0)	0.420
Renal insufficiency, *n* (%)	65 (19.2)	17 (20.0)	48 (19.0)	0.835
Anticoagulation, *n* (%)	38 (11.2)	7 (8.2)	31 (12.3)	0.310
Previous abdominal surgery, *n* (%)	111 (32.8)	35 (41.2)	76 (30.0)	0.059
Clinical presentation				
Acute cholangitis, *n* (%)	103 (30.5)	22 (25.9)	81 (32.0)	0.288
Acute cholecystitis, *n* (%)	82 (24.3)	33 (38.8)	49 (19.4)	**0.001**
Biliary pancreatitis, *n* (%)	72 (21.3)	10 (11.8)	62 (24.5)	**0.013**

Values are mean ± standard deviation or *n* (%)

Bold *p*-values indicate statistical significance (*p* < 0.05)

*P*-values refer to the comparison between *eCCY* and *dCCY* groups

*BMI* body mass index, *ASA* American Society of Anesthesiologists classification, *eCCY* early cholecystectomy (≤ 14 days), *dCCY* delayed cholecystectomy (> 14 days)

The overall median stent dwell time was 77 days (IQR 60–94). Median stent dwell time was 70 days (IQR 55–84) in the eCCY group and 80 days (IQR 62–99) in the dCCY group.

### Recurrent biliary events

With regard to RBE, no statistically significant difference was observed between the eCCY and dCCY groups (17 patients [20.0%] vs. 40 patients [15.8%], *p* = 0.372) (Table 2).

**Table 2:** Clinical outcomes of patients undergoing early versus delayed cholecystectomy after ERCP with biliary stenting

Outcomes	eCCY (*n* = 85)	dCCY (*n* = 253)	*p*-value
Recurrent biliary events (RBE), *n* (%)	17 (20.0)	40 (15.8)	0.372
Stent dysfunction	2	3	–
Acute cholecystitis	9	28	–
Cholangitis	0	3	–
Biliary pancreatitis	0	0	–
Persistent/recurrent abdominal pain after clearance	4	2	–
Choledocholithiasis	2	4	–
Interval-related outcomes			
Time from ERCP to surgery, days, median (IQR)	4 (2–8)	43 (29–64)	–
Operative outcomes			
Operative time, min, median (IQR)	82 (64–114)	86 (61–110)	0.744
Conversion to open surgery, *n* (%)	11 (12.9)	23 (9.1)	0.307
Blood loss, mL, median (IQR)	0 (0–150)	0 (0–150)	0.751
Postoperative outcomes			
Clinically relevant complications (CD ≥ III), *n* (%)	7 (8.2)	32 (12.6)	0.271
Reoperation, *n* (%)	1 (1.2)	8 (3.2)	0.325
Postoperative length of stay, days, median (IQR)	3 (2–6)	3 (2–5)	0.102
Total length of hospital stay, days, median (IQR)	12 (8–16)	13 (10–18)	**0.023**

Values are median (*IQR*) or *n* (%)

Bold *p*-values indicate statistical significance (*p* < 0.05)

*IQR* interquartile range, *min* minutes, *mL* milliliters, *CD* Clavien–Dindo classification, *eCCY* early cholecystectomy, *dCCY* delayed cholecystectomy, *RBE* recurrent biliary event, *ERCP* endoscopic retrograde cholangiopancreatography

The most common RBE was acute cholecystitis, with an overall incidence of 10.9% in the study population, accounting for 64.9% of all RBEs. In the eCCY group, nine patients developed acute cholecystitis that was not present at the time of initial presentation. Stent dysfunction occurred in five patients and consisted of stent occlusion in one patient and stent migration in four patients. Other RBE included persistent abdominal pain after ERCP and recurrent choledocholithiasis (Table 2).

When applying an objective RBE definition excluding the less specific endpoint of persistent or recurrent abdominal pain after ERCP with stenting, 51 patients (15.1%) experienced an objectively verified RBE. Objective RBE rates were virtually identical between groups in the unweighted analysis (15.3% eCCY vs. 15.0% dCCY, *p* = 0.951). These results are summarized in Supplementary Table S6.

#### Multivariable logistic regression analysis

In multivariable logistic regression analysis, timing of cholecystectomy was not associated with the occurrence of recurrent biliary events (OR 1.40, 95% CI 0.71–2.76, *p* = 0.340). Increasing age was associated with a reduced risk of RBE (OR 0.96 per year, 95% CI 0.95–0.98, *p* < 0.001).

Biliary pancreatitis at presentation was associated with a lower likelihood of RBE (OR 0.40, 95% CI 0.16–0.99, *p* = 0.048), while acute cholecystitis showed a trend toward a reduced risk that did not reach statistical significance (OR 0.44, 95% CI 0.19–1.01, *p* = 0.054). Cholangitis was not associated with RBE (*p* = 0.367).

Multivariable regression confirmed that timing was also not independently associated with objective RBE (OR 0.86, 95% CI 0.38–1.93, *p* = 0.715; Supplementary Table S7).

#### Incidence rate per person-time

The cumulative observation time was 559 person-days in the eCCY group (median 4 days per patient, IQR 2–8) and 18,104 person-days in the dCCY group (median 43 days per patient, IQR 29–64). The incidence rate of RBE was 3.04 per 100 person-days (95% CI 1.89–4.89) in the eCCY group and 0.22 per 100 person-days (95% CI 0.16–0.30) in the dCCY group. The results of the incidence rate per person-time analysis is presented in Supplementary Table S2.

#### Time-to-event analysis

**Fig. 2:**
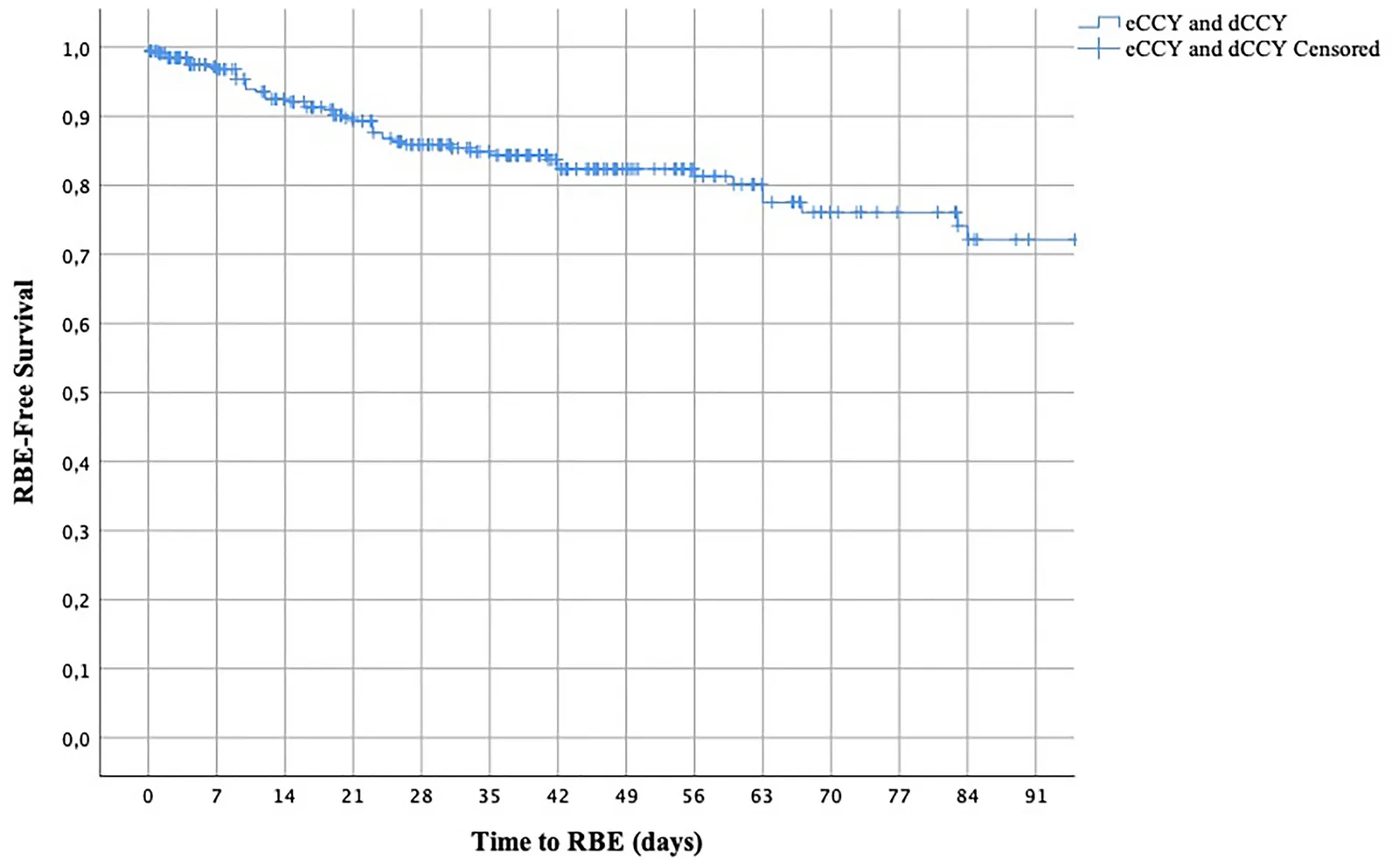
Kaplan–Meier curve showing the cumulative recurrent biliary event-free survival in the overall cohort

Kaplan–Meier analysis demonstrated that RBE predominantly occurred within the early period following ERCP, with cumulative incidence increasing only slightly thereafter. The analysis demonstrated a significant difference in RBE-free survival between groups (log-rank *p* < 0.001) (Fig. 2). Events were predominantly observed within the early post-ERCP period, as reflected by a rapid initial decline in RBE-free survival, followed by a subsequent plateau. Overall, the majority of patients remained free of recurrent biliary events throughout the bridging period (Fig. 2). A supplementary Cox regression analysis is presented in Supplementary Table S3.

### Intraoperative outcomes

There were no statistically significant differences in intraoperative outcomes between the two groups. Median operative time was 82 min (IQR 64–114) in the eCCY group and 86 min (IQR 61–110) in the dCCY group (*p* = 0.744). Conversion to open surgery occurred in 12.9% of patients in the eCCY group and 9.1% in the dCCY group (*p* = 0.307). Estimated blood loss was minimal in both groups (0 mL [IQR 0–150] vs. 0 mL [IQR 0–150], *p* = 0.751) (Table 2).

### Postoperative outcomes

**Fig. 3:**
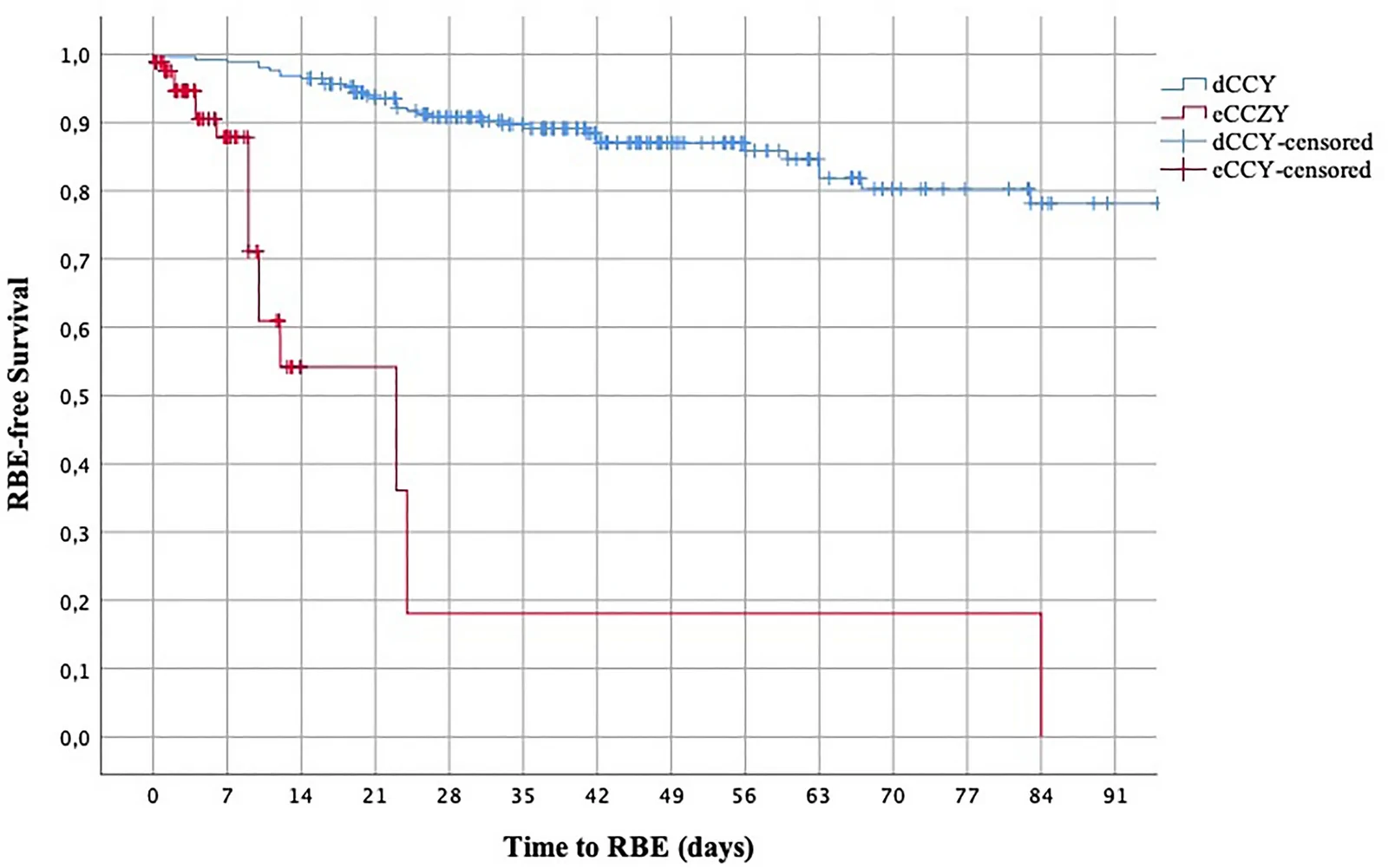
Kaplan–Meier curve showing the cumulative recurrent biliary event-free survival in patients in the eCCY and dCCY group separately. Log-rank *p* < 0.001

Postoperative outcomes were also comparable between both groups. Clinically relevant complications (Clavien–Dindo ≥ III) occurred in 11.5% of the overall cohort, including 8.2% in the eCCY group and 12.6% in the dCCY group (*p* = 0.271). Median postoperative length of stay was similar in both groups (3 days [IQR 2–6] vs. 3 days [IQR 2–5], *p* = 0.102) (Fig. 3).

However, the total length of hospital stay was significantly shorter in the eCCY group compared with the dCCY group (12 days [IQR 8–16] vs. 13 days [IQR 10–18], *p* = 0.023) (Table 2).

### Bridging failure

Among the 253 patients in whom elective interval cholecystectomy was planned after bile duct clearance, 40 patients developed a recurrent biliary event and 33 required urgent cholecystectomy during the bridging period.

In the subgroup analysis comparing patients undergoing urgent surgery due to RBE with those undergoing early elective cholecystectomy (eCCY), there was no statistically significant difference in the operative time (96 min [IQR 81–115] vs. 82 min [IQR 64–114], *p* = 0.134) or in the rate of conversion to open surgery (24.2 vs. 12.9%, *p* = 0.134). However, estimated intraoperative blood loss was significantly higher in the urgent surgery group (200 mL [IQR 0–300] vs. 0 mL [IQR 0–150], *p* = 0.002).

Regarding postoperative outcomes, clinically relevant complications (Clavien–Dindo ≥ III) occurred more frequently in patients undergoing urgent surgery compared with the eCCY group but were not statistically significant (21.2 vs. 8.2%, *p* = 0.062). Reoperation rates were comparable between both groups (6.1 vs. 1.2%, *p* = 0.130) (Table 3).

**Table 3:** Multivariable logistic regression analysis for recurrent biliary events

Variable	OR	95% CI	*p*-value
Timing of cholecystectomy (eCCY vs. dCCY)	1.40	0.71–2.76	0.340
Age (per year)	0.96	0.95–0.98	** < 0.001**
Acute cholecystitis	0.44	0.19–1.01	0.054
Cholangitis	1.38	0.70–2.74	0.367
Biliary pancreatitis	0.40	0.16–0.99	**0.048**

Bold *p*-values indicate statistical significance (*p* < 0.05)

*OR* odds ratio, *CI* confidence interval, *eCCY* early cholecystectomy, *dCCY* delayed cholecystectomy

Postoperative length of stay tended to be longer in the urgent surgery group (5 days [IQR 3–8] vs. 3 days [IQR 2–6], *p* = 0.068), although this difference did not reach statistical significance. In contrast, the total length of hospital stay was significantly longer in patients requiring urgent surgery (15 days [IQR 12–23] vs. 12 days [IQR 8–16], *p* = 0.002). Detailed results of the subgroup analysis are presented in Table 4.

**Table 4:** Intra- and postoperative outcomes of patients undergoing urgent versus early cholecystectomy

Outcomes	Urgent surgery (*n* = 33)	eCCY (*n* = 85)	*p*-value
Operative outcomes			
Operative time, min, median (IQR)	96 (81–115)	82 (64–114)	0.134
Conversion to open surgery, n (%)	8 (24.2)	11 (12.9)	0.134
Estimated blood loss, mL, median (IQR)	200 (0–300)	0 (0–150)	**0.002**
Postoperative outcomes			
Clinically relevant complications (CD ≥ III), *n* (%)	7 (21.2)	7 (8.2)	0.062
Reoperation, *n* (%)	2 (6.1)	1 (1.2)	0.130
Postoperative length of stay, days, median (IQR)	5 (3–8)	3 (2–6)	0.068
Total length of stay, days, median (IQR)	15 (12–23)	12 (8–16)	**0.002**

Values are median (*IQR*) or *n* (%)

Bold *p*-values indicate statistical significance (*p* < 0.05)

*eCCY* early cholecystectomy, *CD* Clavien–Dindo classification, *IQR* interquartile range, *min* minutes, *mL* milliliters

*Inverse probability of treatment weighting (IPTW)* To account for baseline imbalances between groups, an IPTW analysis was performed. After weighting, all covariates were adequately balanced between groups, with all SMD below 0.1 except for anticoagulation (SMD 0.102), which was considered negligible (Supplementary Table S1). In the IPTW-weighted cohort, RBE occurred in 19.8% of patients in the eCCY group and 16.6% in the dCCY group (*p* = 0.503; Table 5). Logistic regression in the weighted cohort confirmed that timing of cholecystectomy was not associated with RBE (OR 1.20, 95% CI 0.64–2.25, *p* = 0.566). Objective RBE remained non-significant in the IPTW-weighted cohort (14.0 vs. 15.8%, *p* = 0.680). IPTW-weighted intraoperative and postoperative outcomes were comparable between groups, including operative time (median 81 vs. 86 min, *p* = 0.784), estimated blood loss (median 0 vs. 0 mL, *p* = 0.145), conversion to open surgery (10.6 vs. 8.7%, *p* = 0.601), clinically relevant complications (Clavien–Dindo ≥ III; 8.1 vs. 12.3%, *p* = 0.296), and postoperative length of stay (median 3.1 vs. 3.0 days, *p* = 0.287). Notably, total length of hospital stay, which was significantly different in the unadjusted analysis (*p* = 0.023), was no longer significant after IPTW adjustment (*p* = 0.054).

**Table 5:** Clinical outcomes of patients undergoing early versus delayed cholecystectomy after ERCP with biliary stenting after inverse probability of treatment weighting (IPTW)

Outcomes	Weighted eCCY	Weighted dCCY	*p*-value
Recurrent biliary events (RBE), *n* (%)	17 (19.8)	42 (16.6)	0.503
Operative outcomes			
Operative time, min, median	81	86	0.784
Conversion to open surgery, *n* (%)	9 (10.6)	22 (8.7)	0.601
Blood loss, mL, median	0	0	0.145
Postoperative outcomes			
Clinically relevant complications (CD ≥ III), *n* (%)	7 (8.1)	31 (12.3)	0.296
Reoperation, *n* (%)	1 (1.2)	9 (3.6)	0.262
Postoperative length of stay, days, median	3.1	3.0	0.287
Total length of hospital stay, days, median	12.8	12.6	0.054

Weighted counts and percentages are shown after application of stabilized *IPTW* weights. Values are median or *n* (%) *IPTW* inverse probability of treatment weighting

Continuous variables compared using weighted Mann–Whitney *U* test; categorical variables using weighted chi-square test

*eCCY* early cholecystectomy, *dCCY* delayed cholecystectomy, *RBE* recurrent biliary event, *CD* Clavien–Dindo classification, *min* minutes, *Ml* milliliters

### Sensitivity analysis: exclusion of patients with acute cholecystitis at presentation

In order to address the most important baseline imbalance between groups, the significantly higher rate of acute cholecystitis at presentation in the eCCY group, a sensitivity analysis was performed excluding all patients with acute cholecystitis at initial diagnosis.

After exclusion of 82 patients presenting with acute cholecystitis at initial diagnosis, 256 patients remained for analysis. The primary findings were unchanged: timing of cholecystectomy was not independently associated with overall RBE or objective RBE, and intraoperative and postoperative outcomes remained comparable between groups. Detailed results are provided in Supplementary Tables S4, S5, S6 and S7. These findings together with the results of the weighted IPTW analysis confirm that the primary results are robust and not driven by the baseline imbalance in acute cholecystitis between groups. The absence of a significant association between timing and both recurrent biliary events and operative outcomes persisted after exclusion of the most important confounder.

## Discussion

In this retrospective single-center study evaluating biliary stenting as a bridging strategy after ERCP for choledocholithiasis, delayed elective cholecystectomy was feasible in selected patients and was not associated with worse intraoperative difficulty or postoperative outcomes compared with early surgery. Although RBE occurred in a subset of patients during the bridging period, their overall incidence and clinical impact were comparable between groups. Delayed surgery was associated with a modest increase in cumulative hospital stay. Notably, patients requiring urgent cholecystectomy due to RBE demonstrated signs of more complex operative conditions.

The role of systematic biliary stenting in patients undergoing delayed cholecystectomy after ERCP remains insufficiently studied. Most previous studies evaluating the timing of cholecystectomy include heterogeneous patient populations in which biliary stenting is inconsistently applied or not reported at all. Consequently, it remains unclear whether adequate biliary drainage through stent placement may mitigate the risks traditionally associated with delayed surgery. From a pathophysiological perspective, temporary biliary stenting may reduce the risk of recurrent biliary obstruction by ensuring continuous bile duct drainage and preventing stone migration or sludge accumulation.

In our cohort, RBEs occurred in 16.9% of our study population, which is within the range reported in the literature. In contrast to previously reported studies, the occurrence of RBE in our cohort did not differ significantly between the eCCY and dCCY groups (20 vs. 15.8%, *p* = 0.372). Furthermore, the RBE rate observed in the dCCY group was markedly lower than the 24–47% reported in earlier studies [2–6, 8]. These differences may be explained by the systematic use of biliary stenting during the waiting period but may also reflect patient selection, successful endoscopic management and established follow-up pathways. However, the independent contribution of biliary stenting itself cannot be determined from the present data, because no non-stented comparator group was available.

Evidence regarding the protective role of biliary stenting remains inconclusive. A recent systematic review and meta-analysis evaluating the potential role of prophylactic biliary stent placement in preventing recurrent biliary events included a total of 755 patients awaiting cholecystectomy [10]. In this analysis, the pooled odds ratio for RBE in patients with biliary stenting was 0.75 (95% CI 0.31–1.79; *I*^2^ = 67%), suggesting no statistically significant protective effect of stent placement. However, the evidence included in this meta-analysis was limited, underpowered and heterogeneous. Of the four studies included, only two were randomized controlled trials, both with relatively small sample sizes. The study by Hormati et al. included 40 patients with biliary pancreatitis and demonstrated a reduction in RBE and gallstone-related complications in the delayed surgery group, suggesting a potential protective effect of biliary stenting [11]. In contrast, the randomized trial by Sasani et al. did not observe a difference in the recurrence rate of choledocholithiasis between groups [12]. Due to the limited number of randomized trials, two retrospective studies were also included in the meta-analysis. While the study by Verzhbitsky et al. found no significant difference in the rate of emergency cholecystectomy or RBE, the larger study by Choi et al., which included more than 400 patients, demonstrated that biliary stenting significantly reduced the rate of bile duct stone recurrence and facilitated clinical recovery [13, 14]. Taken together, the available evidence remains inconclusive and is characterized by substantial heterogeneity.

In our cohort, acute cholecystitis was the most common recurrent biliary event (64.9%), whereas stent dysfunction, persistent abdominal pain, and recurrent choledocholithiasis occurred less frequently.

Another important finding of this study is that delaying cholecystectomy in patients with biliary stent placement did not seem to increase operative difficulty or postoperative morbidity. Operative time, conversion to open surgery, and postoperative complication rates were similar between the eCCY and dCCY groups. The conversion rates in the literature are heterogeneous ranging between 8 and 55% in the late cholecystectomy group [2–4, 6, 8, 15–17].

The potential impact of biliary stenting on subsequent cholecystectomy outcomes has also been explored in the literature, with conflicting results. Nair et al. reported that the presence of a bile duct stent for six weeks prior to elective laparoscopic cholecystectomy influenced several operative parameters, including operative time, conversion to open surgery, bile leak rate, and length of hospital stay [18]. These findings have raised concerns that the presence of a biliary stent may increase surgical difficulty, potentially due to local inflammation or fibrosis around the bile duct. However, other studies have not confirmed these observations and reported no association between biliary stent placement and adverse surgical outcomes. In these reports, biliary stenting was not identified as a predictor of conversion to open surgery or prolonged operative time [13, 19, 20].

The findings of the present study support the latter observations. In our cohort, delayed cholecystectomy in patients with biliary stent placement was not associated with increased operative time, conversion rates, or postoperative morbidity.

Given the retrospective design and non-random treatment allocation, patients undergoing early and delayed cholecystectomy differed in baseline characteristics, introducing potential confounding by indication. Patients in the early cholecystectomy group more frequently presented with acute cholecystitis (38.8 vs. 19.4%, *p* = 0.001), whereas biliary pancreatitis was more frequently observed in the delayed group (24.5 vs. 11.8%, *p* = 0.013). This indicates that treatment allocation may have been influenced by clinical judgment. To address this limitation, we employed three complementary analytical approaches. First an IPTW analysis based on clinically relevant baseline variables. IPTW substantially improved covariate balance, and the weighted outcome analyses confirmed the absence of significant differences in RBE and perioperative outcomes (Table 5). Second, a multivariable logistic regression adjusting for baseline clinical differences demonstrated that timing of cholecystectomy was not independently associated with RBE. And finally, a sensitivity analysis excluding patients with acute cholecystitis confirmed all primary findings.

A methodological consideration in this study is the substantial difference in observation time between the two groups, with a median of 4 days in the eCCY group compared with 43 days in the dCCY group. To provide additional context beyond the crude event proportions, we calculated incidence rates per person-time. The incidence rate was notably higher in the eCCY group (3.04 per 100 person-days vs. 0.22 per 100 person-days), reflecting the concentration of events within a short observation window in a clinically higher-risk subgroup. Similarly, a supplementary Cox regression analysis demonstrated a significantly higher hazard of RBE in the eCCY group (HR 11.55, 95% CI 5.61–23.78, Supplementary Table S1). Both of these findings are best explained by non-random treatment allocation: patients perceived to be at higher risk of early recurrence, particularly those with acute cholecystitis, were preferentially selected for early surgery. The higher incidence rate and hazard in the eCCY group therefore reflect the baseline risk profile of these patients, not a detrimental effect of early surgery.

This interpretation is supported by the sensitivity analysis excluding patients with acute cholecystitis at presentation, which represented the most important baseline imbalance between groups (38.8 vs. 19.4%, *p* = 0.001). In the restricted cohort of 256 patients, the primary findings were confirmed: timing of cholecystectomy was not independently associated with RBE, and intraoperative and postoperative outcomes remained comparable between groups. These results strengthen the conclusion that the comparable outcomes observed in the full cohort are not attributable to confounding by acute cholecystitis and that delayed cholecystectomy with biliary stenting does not confer an increased risk of adverse outcomes.

The observed association between biliary pancreatitis and a lower risk of RBE (OR 0.40, 95% CI 0.16–0.99, *p* = 0.048) likely reflects differences in treatment allocation and clinical pathways rather than a true protective effect.

The RBE definition included both objectively verifiable events and the less specific endpoint of persistent or recurrent abdominal pain. While this broad definition may capture clinically relevant symptoms that prompt medical evaluation, it may also introduce classification bias. A sensitivity analysis using a restrictive definition limited to objectively verifiable events yielded virtually identical RBE rates between groups (15.3 vs. 15.0%, *p* = 0.951; Supplementary Table S5).

Taken together, these findings suggest that delayed cholecystectomy after biliary stenting does not confer an increased overall risk of RBE when compared to early surgery after biliary stenting, but rather that early events occur in a subset of patients with higher baseline risk. The risk of RBE is highest in the early phase after ERCP, while most patients remain event-free thereafter.

Although delayed surgery appeared safe in most patients, our data also highlight the consequences of bridging failure. In our cohort, 220 patients (86.9%) underwent elective cholecystectomy as planned, whereas 40 patients were readmitted due to RBE and 33 ultimately required urgent cholecystectomy.

In the subgroup analysis of patients undergoing urgent surgery, operative time was not significantly different compared with the early cholecystectomy group (96 min [IQR 81–115] vs. 82 min [IQR 64–114], *p* = 0.134). Although a median blood loss of 200 mL may still be considered clinically modest, it was significantly higher compared with the early surgery group. Moreover, the conversion rate to open surgery was not statistically different but was numerically almost twice as high in patients undergoing urgent surgery compared with those undergoing early elective cholecystectomy (24.2 vs. 12.9%, *p* = 0.134). Similarly, clinically relevant complications (Clavien–Dindo ≥ III) tended to occur more frequently in patients undergoing urgent surgery compared with the eCCY group (21.2 vs. 8.2%, *p* = 0.062).

These findings highlight that in selected patients, delayed surgery may be feasible. However, patients who develop RBE during the waiting period represent a clinically relevant subgroup with potentially increased operative complexity. Identifying patients at increased risk for bridging failure may therefore represent an important step in optimizing treatment strategies.

Overall, our findings suggest that, in selected patients, biliary stenting may represent a safe and pragmatic management strategy that does not compromise operative outcomes when early surgery is not feasible. This interpretation is limited to a stented cohort and does not establish the efficacy of biliary stenting as a protective intervention. The main disadvantage of delayed surgery in our cohort was a modest increase in cumulative hospital stay and the moderate risk of necessity of an urgent cholecystectomy with potentially increased conversion and complication rate.

## Limitations

Several limitations must be considered when interpreting the results of this study. First, the retrospective design introduces the potential for selection bias and confounding by indication. Although IPTW and a sensitivity analysis excluding patients with acute cholecystitis were used to improve baseline comparability and reduce measured confounding, residual confounding from unmeasured variables cannot be excluded. Second, this was a single-center study, which may limit the generalizability of the results to other institutions or healthcare systems. Third, although the overall cohort size was substantial, the group sizes were imbalanced (85 vs. 253 patients), which may affect statistical power for detecting smaller differences, particularly in subgroup analyses. Fourth, the absence of a non-stented comparator group precludes conclusions regarding the protective effect of biliary stenting itself; the present study evaluates the safety of delayed cholecystectomy in the presence of a stent, not the efficacy of stenting per se. Fifth, some ERCP-related variables, including stent length, stone size, stone number, and common bile duct diameter, were not consistently documented and could not be included in the analysis. Furthermore, unmeasured confounders, including variations in surgical expertise and individual decision-making regarding the timing of surgery may have influenced treatment allocation and outcomes. Finally, this study did not assess the economic or environmental impact of the strategy. Biliary stent placement inherently necessitates a subsequent ERCP for stent removal, thereby increasing procedural volume, resource utilization, and healthcare costs.

## Conclusion

In this retrospective cohort of patients undergoing ERCP with biliary stent placement for choledocholithiasis, delayed elective cholecystectomy appears to be feasible in selected patients and was not associated with worse perioperative outcomes compared with early cholecystectomy. These findings remained consistent after IPTW adjustment and when using a stricter definition of objective RBE. However, due to the absence of a non-stented control group and the risk of residual confounding, this study cannot determine whether biliary stenting itself reduces recurrent biliary events. Delayed cholecystectomy after biliary stenting may therefore represent a pragmatic management option when early surgery is not feasible. Patients requiring urgent surgery while waiting for elective cholecystectomy due to RBE during the waiting period represent a higher-risk subgroup, and future studies should focus on identifying patients at increased risk of bridging failure.

## Supplementary Information

Below is the link to the electronic supplementary material.

Supplementary file1 (DOCX 18 kb)
