# Supplementary material for: Biliary stenting as a bridge to cholecystectomy in patients with choledocholithiasis
Source: Surg Endosc. 2026 Aug 21;40(11):9852–64. doi: 10.1007/s00464-026-13260-z (PMC13754946; doi:10.1007/s00464-026-13260-z)
Supplement: Supplementary file 1 [file 464_2026_13260_MOESM1_ESM.docx]

## **Supplementary Tables**

Supplementary Table S1 - Covariate balance before and after IPTW

| Variable | SMD before IPTW | SMD after IPTW |
| --- | --- | --- |
| Age | 0.100 | 0.083 |
| Male sex | 0.222 | 0.040 |
| BMI ≥30 kg/m² | 0.030 | 0.057 |
| ASA I | 0.140 | 0.037 |
| ASA II | 0.055 | 0.026 |
| ASA III | 0.080 | 0.000 |
| ASA IV | 0.095 | 0.007 |
| Renal insufficiency | 0.026 | 0.025 |
| Anticoagulation | 0.133 | 0.102 |
| Previous abdominal surgery | 0.234 | 0.047 |
| Acute cholangitis | 0.136 | 0.004 |
| Acute cholecystitis | 0.439 | 0.040 |
| Biliary pancreatitis | 0.335 | 0.074 |

*Absolute standardized mean differences are shown. Values <0.1 were considered indicative of adequate balance. IPTW – inverse probability of treatment weighting; SMD – standardized mean difference.*

Supplementary Table S2 - Incidence rates of recurrent biliary events per person-time

| Outcomes | eCCY (n=85) | dCCY (n=253) | Total (n=338) |
| --- | --- | --- | --- |
| Number of RBE | 17 | 40 | 57 |
| Cumulative observation time, person-days | 559 | 18,104 | 18,663 |
| Mean observation time per patient, days | 6.6 | 71.6 | 55.2 |
| Incidence rate per 100 person-days (95% CI) | 3.04 (1.89–4.89) | 0.22 (0.16–0.30) | 0.31 (0.24–0.40) |

*RBE – recurrent biliary event; CI – confidence interval; eCCY – early cholecystectomy; dCCY – delayed cholecystectomy 95% confidence intervals calculated using the exact Poisson method*

Supplementary Table S3 - Multivariable Cox regression analysis for time to recurrent biliary events

| Variable | HR | 95% CI | p-value |
| --- | --- | --- | --- |
| Timing of cholecystectomy (eCCY vs. dCCY) | 11.55 | 5.61–23.78 | **<0.001** |
| Age (per year) | 0.96 | 0.95–0.98 | **<0.001** |
| Acute cholecystitis | 0.86 | 0.41–1.80 | 0.696 |
| Cholangitis | 1.05 | 0.58–1.92 | 0.885 |
| Biliary pancreatitis | 0.49 | 0.20–1.22 | 0.110 |

*HR – hazard ratio; CI – confidence interval; eCCY – early cholecystectomy; dCCY – delayed cholecystectomy Bold p-values indicate statistical significance (p < 0.05) Note: The significantly elevated hazard ratio for early cholecystectomy most likely reflects non-random treatment allocation (confounding by indication) rather than a causal effect of surgical timing. See Discussion for detailed interpretation.*

Supplementary Table S4 - Clinical outcomes excluding patients with acute cholecystitis at presentation

| **Outcomes** | **eCCY (n=52)** | **dCCY (n=204)** | **p-value** |
| --- | --- | --- | --- |
| Recurrent biliary events (RBE), n (%) | 14 (26.9) | 35 (17.2) | 0.110 |
| Stent dysfunction | 1 | 3 | – |
| Acute cholecystitis | 8 | 23 | – |
| Cholangitis | 0 | 3 | – |
| Biliary pancreatitis | 0 | 0 | – |
| Persistent / recurrent abdominal pain | 4 | 2 | – |
| Choledocholithiasis | 1 | 4 | – |
| Operative outcomes |  |  |  |
| Operative time, min, median (IQR) | 90 (62–118) | 85 (60–111) | 0.407 |
| Conversion to open surgery, n (%) | 6 (11.5) | 18 (8.8) | 0.549 |
| Blood loss, mL, median (IQR) | 0 (0–100) | 0 (0–100) | 0.322 |
| Postoperative outcomes |  |  |  |
| Clinically relevant complications (CD ≥ III), n (%) | 5 (9.6) | 26 (12.7) | 0.537 |
| Reoperation, n (%) | 1 (1.9) | 8 (3.9) | 0.986 |
| Postoperative length of stay, days, median (IQR) | 3 (2–5) | 3 (2–5) | 0.356 |
| Total length of hospital stay, days, median (IQR) | 13 (9–16) | 13 (9–18) | 0.358 |

*Values are median (IQR) or n (%) IQR – interquartile range; min – minutes; mL – milliliters; CD – Clavien–Dindo classification; eCCY – early cholecystectomy; dCCY – delayed cholecystectomy; RBE – recurrent biliary event Note: Patients with acute cholecystitis at initial presentation were excluded (n = 82)*

Supplementary Table S5- Multivariable logistic regression analysis for recurrent biliary events, excluding patients with acute cholecystitis at presentation

| Variable | OR | 95% CI | p-value |
| --- | --- | --- | --- |
| Timing of cholecystectomy (eCCY vs. dCCY) | 1.71 | 0.81–3.63 | 0.159 |
| Age (per year) | 0.96 | 0.94–0.98 | **<0.001** |
| Cholangitis | 1.58 | 0.72–3.44 | 0.254 |
| Biliary pancreatitis | 0.39 | 0.14–1.06 | 0.065 |

*OR – odds ratio; CI – confidence interval; eCCY – early cholecystectomy; dCCY – delayed cholecystectomy Note: Patients with acute cholecystitis at initial presentation were excluded (n = 82). Acute cholecystitis was not included as a covariate. Bold p-values indicate statistical significance (p < 0.05)*

Supplementary Table S6 – Sensitivity analysis using objective RBE definition

| Analysis | eCCY | dCCY | OR (95% CI) | p-value | |
| --- | --- | --- | --- | --- | --- |
| Full cohort (n = 338) |  |  |  | |  |
| Unweighted | 13/85 (15.3) | 38/253 (15.0) | 1.02 (0.52–2.02) | | 0.951 |
| IPTW-weighted | 12/86 (14.0) | 40/253 (15.8) | 1.13 (0.57–2.27) | | 0.680 |
| Excluding cholecystitis (n = 256) |  |  |  | |  |
| Unweighted | 10/52 (19.2) | 33/204 (16.2) | 1.23 (0.56–2.70) | | 0.599 |

*RBE – recurrent biliary event; OR – odds ratio; CI – confidence interval; eCCY – early cholecystectomy; dCCY – delayed cholecystectomy; IPTW – inverse probability of treatment*

Supplementary Table S7 – Multivariable logistic regression for objective RBE

|  |  | Full cohort (n = 338) | | | Excl. cholecystitis (n = 256) | | |
| --- | --- | --- | --- | --- | --- | --- | --- |
| Variable |  | OR | 95% CI | p | OR | 95% CI | p |
| Timing of cholecystectomy (eCCY vs. dCCY) |  | 0.98 | 0.47–2.02 | 0.955 | 0.86 | 0.38–1.93 | 0.715 |
| Age (per year) |  | 0.97 | 0.95–0.99 | **<0.001** | 0.97 | 0.95–0.99 | **0.001** |
| Acute cholecystitis |  | 0.56 | 0.24–1.29 | 0.170 | – | – | – |
| Cholangitis |  | 1.35 | 0.66–2.78 | 0.408 | 1.55 | 0.70–3.43 | 0.277 |
| Biliary pancreatitis |  | 0.46 | 0.18–1.14 | 0.093 | 0.46 | 0.17–1.26 | 0.133 |

*OR – odds ratio; CI – confidence interval; eCCY – early cholecystectomy; dCCY – delayed cholecystectomy Bold p-values indicate statistical significance (p < 0.05)*
